# Effect of non-contact induction heating on HA coatings and bone cement, an ex vivo study

**DOI:** 10.12688/f1000research.148225.2

**Published:** 2024-10-22

**Authors:** Robert Kamphof, Dr. Giuseppe Cama, Jeroen Mesman-Vergeer, Dr. Rob G.H.H. Nelissen, Dr. Bart G.C.W. Pijls

**Affiliations:** 1 Department of Orthopaedics, Leiden University Medical Center, Leiden, Albinusdreef 2, 2333 ZA, The Netherlands; 2 CAM Bioceramics B.V., Leiden, Zernikedreef 6, 2333 CL, The Netherlands; 3 FMD / Institute LION / Leiden University, Leiden, Niels Bohrweg 2, 2333 CA, The Netherlands

**Keywords:** PJI, non-contact induction heating, AMR, hydroxyapatite, calcium phosphate, implant coating, acrylic bone cement, PMMA, titanium implants

## Abstract

**Background:**

Prosthetic joint infection is a serious complication that can arise after total joint replacement surgery. When bacteria colonise an orthopaedic implant, they form biofilms that protect them from their environment, making them difficult to remove. Treatment is further complicated by a global rise of antimicrobial resistance. These protective mechanisms make treatment of prosthetic joint infection increasingly complex. Non-contact induction heating is an upcoming technology that uses heat to eradicate bacteria that are present on the surface of metallic implants. This study aims to provide insight into the feasibility of using non-contact induction heating on metallic implants that are in direct contact with other biomaterials, such as coatings composed of hydroxyapatite and bone cement composed of poly (methyl methacrylate) (PMMA).

**Methods:**

Characterisation of hydroxyapatite coatings and adhesion strength tests were conducted according to standards set by the International Organisation for Standardisation (ISO 13779-2). The fixation strength of acrylic bone cement was tested according to an adapted method from ISO.

**Results:**

It was found that non-contact induction heating did not significantly affect the adhesion strength of hydroxyapatite coatings. In contrast to hydroxyapatite coatings, acrylic bone cement softened temporarily as the temperature exceeded the glass transition temperature (83.38 ± 10.88°C). However, the induction heating temperature had no significant effect on the fixation strength after the cement was allowed to cool down.

**Conclusion:**

This study shows the feasibility of using non-contact induction heating up to 80°C when bone cement or ceramic coatings are present in contact with infected metallic implants.

## Introduction

One of the adverse outcomes of total joint replacement surgery is prosthetic joint infection (PJI). Commonly caused by gram-positive bacteria, such as S. aureus and S. epidermidis, PJI can have a devastating impact on patients’ lives and comes with a significant financial burden on society. ^1^^,^^2^ Due to the formation of protective biofilms by these pathogens, PJI is difficult to treat using conventional antibiotics. Currently, the gold standard to treat PJI is surgical treatment in combination with antibiotics (DAIR: debridement, antibiotics and implant retention). Despite this extensive procedure, DAIR fails in over 30% of all cases, necessitating further surgery for implant removal and replacement. ^3^ Moreover, the increasing incidence of antimicrobial resistance (AMR) further complicates treatment of PJI using antibiotics. ^4^ As it is expected that the problem of AMR will only increase, our reliance on antibiotics to treat infections is not sustainable long-term. Consequently, different treatment modalities are needed, beyond the ones currently available.

Noncontact induction heating (NCIH) is a novel approach to treating bacterial biofilms. NCIH is a treatment method that is under development to combat PJI. NCIH can be used to deliver localised thermal damage to biofilms on metal implants. ^5^ This method could be used as part of DAIR to kill bacteria in places that are hard to clean manually. Potentially, NCIH could even be used to treat implant-related infection non-invasively. ^6^ Previous studies have shown that NCIH, alone or in combination with mechanical cleaning or antibiotics, is capable of significantly reducing or even eradicating bacterial biofilms on titanium surfaces. ^7^^–^^9^ Notably, it is possible to perform NCIH on existing implants without the need for modification, meaning that metallic implants that are already inside patients today could be eligible for NCIH treatment.

For a favourable clinical outcome, it is essential that an orthopaedic implant remains fixed securely. In that respect, it is important to investigate if NCIH treatment does not lead to implant loosening before NCIH can be applied in the clinic. To address this question, this study investigates the effects of NCIH on the adhesion strength of ceramic implant coatings and acrylic bone cement to the metal implant surface, which are the most frequently used biomaterials in direct contact with metallic implants.

Ceramic implant coatings made of hydroxyapatite (HA) are commonly used on uncemented prostheses. Because of the similarities of the chemical structure of HA with that of natural bone, HA coatings are highly biocompatible. Implants coated with HA have been shown to expedite and improve implant fixation. ^10^ If NCIH is employed on a coated implant, it is critical to ensure that the integrity of the coating is not compromised by the heat generated in the metallic part of the implant. If this were to happen, coating delamination is expected to cause severe third body wear on prosthetic joints.

Polymeric bone cement is used to fixate prosthetic joints during prosthetic joint replacement surgery. These materials are made by mixing precursor materials to make a pliable putty that hardens into solid poly (methyl methacrylate) (PMMA). Like most polymers, PMMA exhibits a glass transition temperature (T _glass_) where the polymer softens considerably. Previous research has shown that bone cement has a glass transition temperature between 80 and 100°C. ^11^ As the prosthetic is held in place by mechanical forces and friction, it is important to investigate the possibility that NCIH treatment loosens the cement’s grip as the temperature approaches T _glass_. As this loosening could happen reversibly or irreversibly, it is important to test the grip strength of bone cement both during and after heating.

Thus, the aim of this study is to investigate the effect of NCIH on the fixation of metallic implants in direct contact with other HA coatings or PMMA bone cement. The research question is ‘does NCIH of Ti6Al4V weaken the fixation of HA coatings or PMMA bone cement?’. Addressing this question is an important step towards the clinical application of NCIH.

## Methods

The effect of different thermal doses on the adhesion of HA coatings and PMMA bone cement was evaluated. The HA coating represents the scenario of uncemented HA coated joint implants. In this scenario, the coating acts as a bioactive cue that supports bone growth to improve implant fixation. It is critical that NCIH does not lead to delamination of the coating. Coating characterisation and adhesion strength tests were conducted according to standards set by the International Organisation for Standardisation (ISO) in ISO-13779-2.

The PMMA bone cement represents the scenario of cemented joint implants. The fixation strength of PMMA was tested during heating as well as after cooling down, as PMMA bone cement exhibits a T _glass_ that is close to the effective range of NCIH. ^11^ When heated at or above T _glass_, the bone cement may become soft, jeopardizing fixation. ^12^ As there are no standards for testing the fixation of acrylic bone cement, the methods used in this study were adapted from the methods used to test the HA coatings.

The raw data for these experiments was posted to the Harvard Dataverse at https://doi.org/10.7910/DVN/C1AWLO.

### HA coatings – adhesion strength of uncemented implants

HA coating powder and substrate materials were provided by CAM Bioceramics B.V., a commercial manufacturer of calcium phosphates. Coating of the metal substrates was also performed by CAM Bioceramics B.V. Grade 5 titanium (Ti6Al4V) cylinders (25 mm diameter, 30 mm length) were coated on one of their circular faces with HA of a thickness of ~50 μm using the plasma spray method. In addition, two Ti6Al4V coupons (38 mm × 25 mm of 1 mm thickness, supplier: https://www.titaniumshop.nl) were also coated.

Prior to coating, the surface to be coated was sandblasted to provide a rough surface for adequate adhesion of the coating. Additional cylinders that were sandblasted, but not HA coated, were used as controls. After plasma spraying, coating thickness was measured using an eddy current device (Fischer ^®^ Fischerscope ^®^ MMS ^®^). Reported coating thickness represents the average of 5 measurements on each coating.

### Physiochemical characterisation of HA-coated Ti6Al4V coupons

Two Ti6Al4V coupons (38 mm × 25 mm of 1 mm thickness, supplier: https://www.titaniumshop.nl) were also coated for physiochemical characterisation of the coating. The titanium material from this supplier was previously characterised in more detail and used for an *in vitro* antimicrobial study. ^13^ The physiochemical properties of the coatings were compared to the requirements for HA coatings for biomedical applications set by ISO 13779-2. ^14^

The surface topography of unmodified Ti6Al4V, sandblasted Ti6Al4V coupons and HA coatings was performed using Scanning Electron Microscopy (SEM) using a Hitachi TM3000. SEM images were taken at various magnifications and at various locations in order to obtain a good impression of the surface topography.

X-ray diffraction (XRD) was used to assess the phase composition and crystallinity ratio of the final coatings. XRD measurements were performed with a Rigaku Miniflex 600. In order to measure the XRD pattern of coated HA, the coating was scraped off the coupon using a scalpel blade. In accordance to ISO 13779-2, the XRD pattern of the scraped coating was measured before and after sintering at 1000°C for 24h to fully crystallise the sample. Sintering was performed using a Nabertherm HT 128 furnace.

XRD patterns were used to obtain the crystallinity ratio and the phase purity according to the method described in ISO 13779-3. ^15^ The phase purity was assessed by qualitatively assessing the presence of peaks not belonging to HA. If such peaks were present, the phase purity was determined by comparing the measured ratio of peak integrals with a calibration curve established by mixing the component phases in a known ratio. The crystallinity ratio was obtained by comparing the peak integrals of 10 peaks known to belong to HA before and after sintering. The XRD measurement for determining the crystallinity ratio was always preceded by measuring the same alumina reference sample. The crystallinity ratio was calculated using the following formula:

$$C=\frac{S1*R2}{S2*R1}*100\%$$

Where S1 is the sum of the integrals of 10 peaks of the uncrystallised sample, S2 is the sum of the integrals of 10 peaks of the fully crystallised sample, R1 is the integral of the main peak of the alumina reference measured before sintering and R2 is the integral of the main peak of the alumina reference measured after sintering.

All XRD patterns were analysed in PDXL2 ( https://www.rigaku.com/support/software/pdxl). PDXL2 is a proprietary software supplied by Rigaku to analyse XRD data generated by Rigaku XRD devices. A free alternative program to analyse XRD data is Profex. ^16^

### Induction heating of HA coated Ti6Al4V cylinders

**Figure 1.**
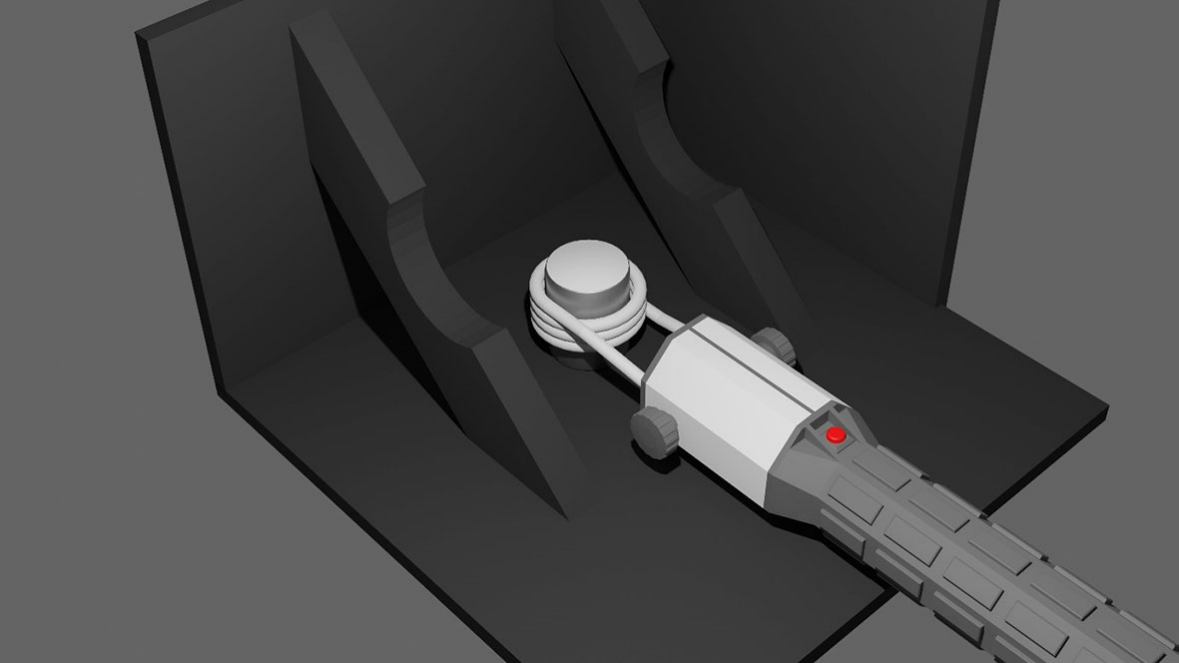
Digital render of the setup for heating the HA coated samples. The portable induction heating device is placed around the coated cylinder. The temperature is monitored using an infrared camera. To minimise interference from the environment, the heating is performed in a custom setup made from wooden panels that are painted black.

Heating of the coated cylinders was performed using a commercially available induction heater (HBM portable induction heater 9305) with a frequency of 45 kHz and a maximal power of 1000W featuring a 3.5cm turn coil of solid copper wire with an inner diameter of 28mm. Heating was applied continuously until the intended temperature was reached. The surface temperature of the HA coating was measured using an IR camera (Testo 872) during induction heating. Each coated cylinder was individually placed inside the coil and heated to the target temperature (see Figure 1). The setup was surrounded by wooden panels painted black in order to limit infrared radiation from the surroundings. The final temperature reached by the centre of the coated surface was recorded. The following groups were considered:

• HA Coated control group without heating (n=6)
• HA Coated group heated to 60°C (n=5)
• HA Coated group heated to 100°C (n=5)
• Uncoated control group (to measure the strength of the glue)

The temperature of 60°C was chosen as a possible temperature to use clinically, as this temperature has been shown to be effective against bacterial biofilms. ^6^^–^^8^^,^^17^ The temperature of 100°C represents a ‘worst case scenario’ that represents the maximum temperature achievable in a wet environment to assess the effect of NCIH on the coating at higher temperatures.

### Adhesion strength tests on HA-coated Ti6Al4V cylinders

Adhesion strength tests were performed according to ISO 13779-4. ^18^ Cylinders were glued together using FM-1000 epoxy adhesive films. Each HA coated cylinder was glued to an uncoated, sandblasted Ti6Al4V cylinder. For control experiments, two sandblasted cylinders without coating were glued to each other. The adhesion strength was measured using a tensile tester.

**Figure 2.**
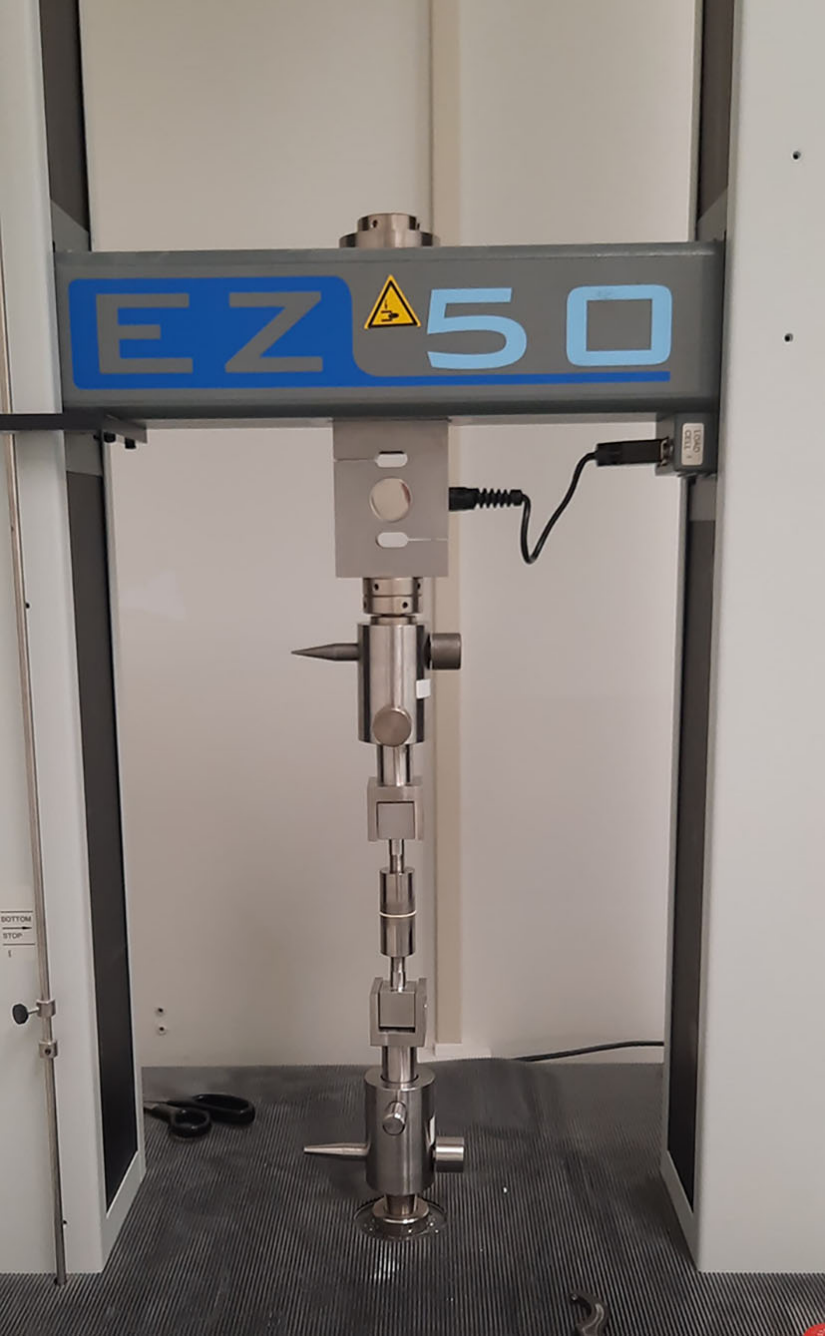
The setup for coating adhesion strength experiments.

Adhesion tests were performed using an EZ50 adhesion tester from Lloyd using a 50kN load cell (see Figure 2). The adhesion strength was defined as the force required to completely separate the two cylinders. Results were recorded in kN and converted to MPa using the formula:

$$\sigma =\frac{F}{A}$$

Where σ is the adhesion strength in Pascal, F is the pull force in Newton, and A is the surface area of the cylinders (491 mm ^2^). The mean adhesion strength and 95% confidence intervals (95% CI) were calculated.

**Figure 3.**
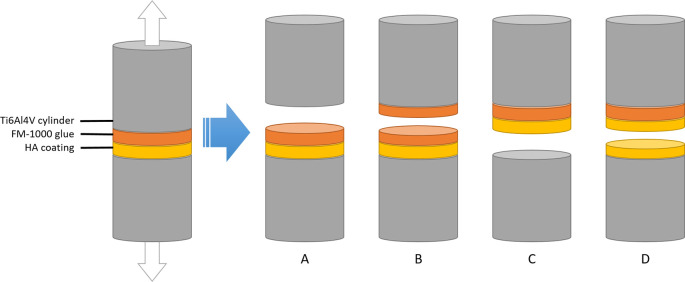
Possible modes of failure for coating adhesion experiments. Between the two cylinders, there is a layer of HA that is plasma-sprayed onto the bottom cylinder, and a layer of fm-1000 glue that is applied by pressing one coated and one coated cylinder together under high temperature. If the cylinders are pulled apart, the setup can fail in 4 different ways depending on which force is the weakest: adhesive forces of the glue (A), cohesive failure of the glue (B), adhesive failure of the coating (C) or cohesive failure of the coating (D). According to ISO-13779-2, only failure modes C and D are acceptable.

After the adhesion tests, the interface between the cylinders was inspected and the mode of failure was recorded. The following modes of failure were considered (see Figure 3):

• Adhesive failure of the glue (A)
• Cohesive failure of the glue (B)
• Adhesive failure of the coating (C)
• Cohesive failure of the coating (D)
• Mixed mode of failure

For control experiments, only failure mode A was considered successful. In line with ISO 13379-4, only failure modes C and D were considered successful for coated samples. Unsuccessful samples were eliminated from further analysis.

### Bone cement – adhesion of cemented implants

To gain more insight into the possible effect of NCIH on the fixation of metal and bone cement, the pull-out force was measured for metal-cement constructs after the exposed metal has been subjected to various temperature regimes by induction heating. The constructs were made to mimic a cemented total hip or knee replacement implant, where part of the implant is covered by the cement and another part is uncovered.

### Sample preparation process

**Figure 4.**
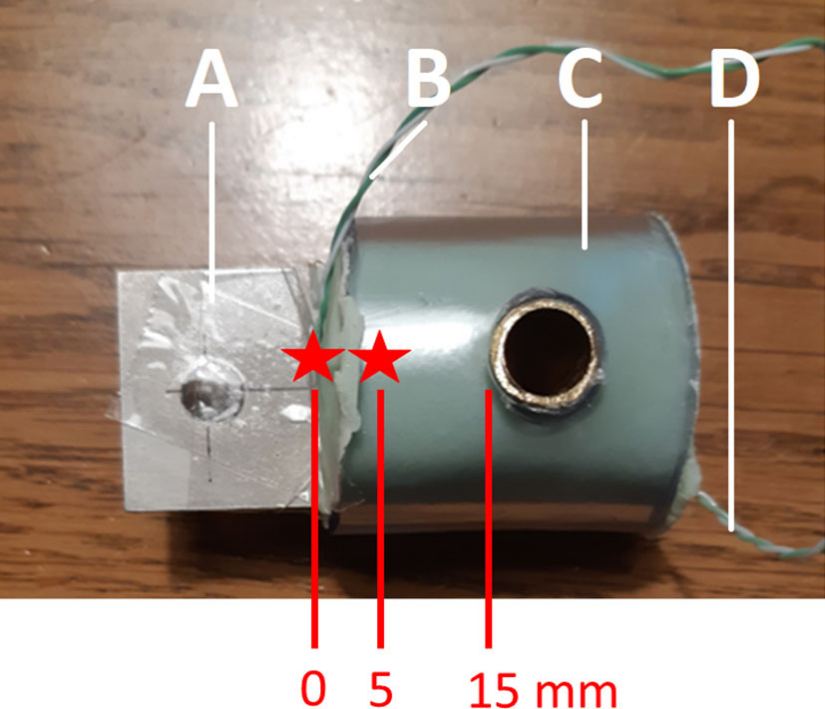
The metal-bone cement construct for pull-out testing. A) the Ti6Al4V coupon inserted 15mm into the bone cement. B) K-type thermocouple attached to the coupon at the air-cement interface for reading the external temperature. C) PMMA bone cement. D) K-type thermocouple attached to the coupon at a depth of 5 mm in the cement for reading the internal temperature. The green cement is mixed and placed inside a transparent plastic tube, through which a copper tube has been drilled. A Ti6Al4V coupon is then inserted into the soft cement, and the cement is allowed to harden. Two thermocouples are attached to the coupon to measure the internal (inside the cement) and external (outside the cement) temperature of the coupon.

To create the metal-cement constructs, the same Ti6Al4V coupons were used that were used for coating with HA. A K-type thermal couple was glued to the metal coupon before inserting the metal into the bone cement at 5 mm depth (see Figure 4). This thermocouple was used to measure the internal temperature of the metal coupon inside the bone cement. Another K-type thermocouple was attached to the metal coupon just outside the metal-cement interface (0 mm into the cement). This thermocouple was used to measure the external temperature of the metal coupon outside the bone cement right at the metal-bone cement interface.

Palacos ^®^ R+G (Hereaus™) bone cement was used for all constructs. This bone cement is a high viscosity bone cement containing gentamicin. It is one of the most frequently used bone cements clinically. ^19^ The metal coupons were inserted into the bone cement at a depth of 15 mm, leaving 23 mm uncovered (see Figure 4). Cement was handled and mixed by hand and pressurization was applied in order to mimic the clinical setting as closely as possible. All constructs were cemented by an orthopaedic surgeon (BP). For each package of bone cement 3 constructs were made. The order in which these constructs were prepared was recorded and used for sensitivity analyses, because timing of insertion during the curing phase (early, medium, late) has been shown to affect pull out strength. ^20^ The relationship between the cement order and the pull-out force was also evaluated.

### Induction heating of cemented Ti6Al4V coupons

Heating of the coated coupons was performed using a custom built induction heater at 150 kHz featuring a 4-turn solenoid heating coil with an inner diameter of 35 mm in a modified Helmholtz configuration: two connected 2-turn solenoid coils (total 4 turns) with 12 mm spacing between them to allow for uniform heating of the metal coupon. A pulsed heating pattern (duty cycle) was applied to ensure a homogeneous heat distribution and to avoid hot spots. Allocation to the heating groups was done randomly, using a random number generator, to minimize the possible bias by cement order or other factors. The following groups were considered:

• Control group, no thermal exposure (n=4)
• Single 70°C heating cycle of the external (not in cement) metal (n=2)
• Single 100°C heating cycle of the external (not in cement) metal (n=3)
• Multiple (n=5) 70°C heating cycles of the external (not in cement) metal (n=3)
• Single 70°C heating cycle of the internal part of the coupon (n=3)

The temperature of 70°C was chosen as a possible temperature to use clinically, as this temperature has been shown to be effective against bacterial biofilms. ^6^^–^^8^^,^^17^ The chosen temperature of 70°C is slightly higher than the 60°C used for HA coated samples, since we expected PMMA to be more effective at insulating the implant, thereby preventing thermal damage to surrounding tissues temperature of 100°C represents a ‘worst case scenario’ to assess the effect of NCIH on the cement at higher temperatures.

### Pull-out experiments on cemented Ti6Al4V coupons

**Figure 5.**
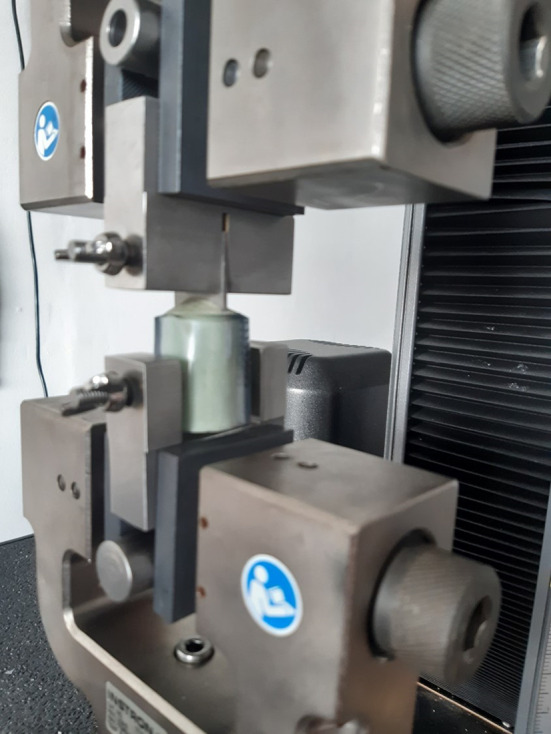
The setup for cement pull-out experiments. The custom-built construct is attached to a tensile tester after heating using an induction heater. The tensile tester then pulls apart the construct and measures the force required to do so.

After heating, a tensile tester (Instron3366) fitted with a 2kN load cell was used to measure the pull-out force of cemented coupons (see Figure 5). The pull-out force (F) was defined as the force required to completely separate the coupon from the cement and was recorded in Newton. The mean pull-out force and 95% CI were calculated.

### *In-situ* induction heating of cemented Ti6Al4V coupons under tension

In order to investigate the strength of PMMA cement under active heating, the samples used for the previous pull-out test were re-used. The Ti6Al4V coupons were re-inserted into the cement using a vise. Subsequently, they were suspended above the ground by a wire and loaded with a weight of 2.5 kg. The coupons were subsequently heated using NCIH until failure. A pulsed heating pattern (duty cycle) was applied to ensure a homogeneous heat distribution and to avoid hot spots. Heating of the coated coupons was performed using a commercially available induction heater (HBM portable induction heater 9305) at 45 kHz with a maximal power of 1000W featuring a 3.5 cm turn coil of solid copper wire with an inner diameter of 28 mm to allow for targeted heating of the cement-metal interface. The internal temperature where the coupon was completely separated from the cement was recorded.

## Results

### HA coatings – adhesion strength of uncemented implants

Detailed data on coating thickness, the recorded maximum heating temperature and mode of failure of individual samples can be found in the Underlying data. ^21^ The average coating thickness was 51.9 μm.

### Physiochemical characterisation of HA-coated Ti6Al4V coupons

**Figure 6.**
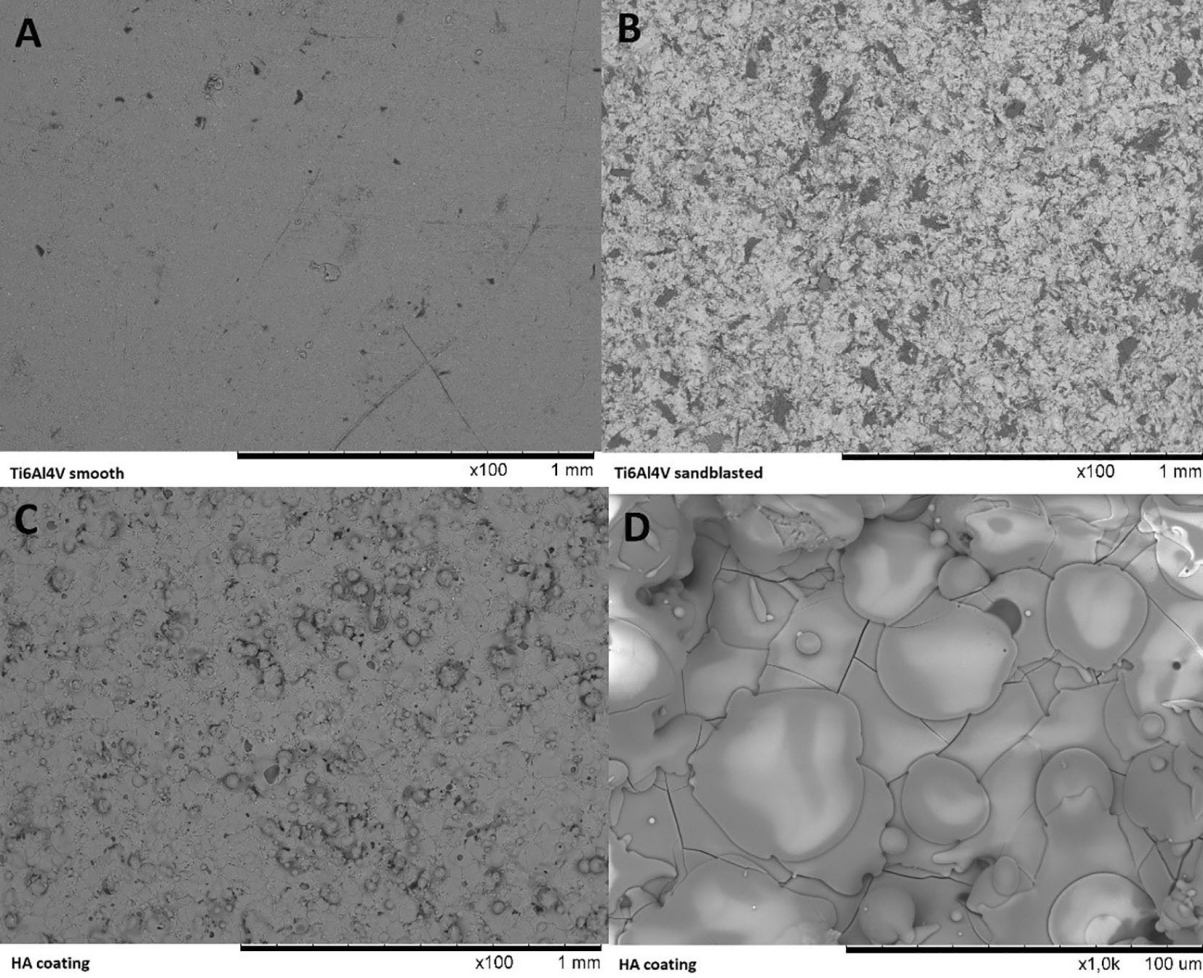
SEM images captured of the surface of a Ti6Al4V coupon before surface treatment (A, 100× magnification), after sandblasting (B, 100× magnification), after coating with HA (C, D, resp. 100× and 1000× magnification).

The SEM images show a clear increase in surface roughness between Ti6Al4V before and after sandblasting (see Figure 6). SEM of the HA coating shows the formation of a continuous and fully covering layer of HA has formed on the metal surface. The surface features of the HA coating show where individual molten particles of HA have struck the surface.

**Figure 7.**
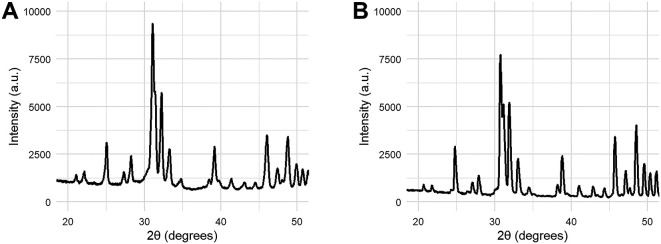
XRD patterns of HA coating scraped from the metal coupon (A) and the same powder after fully crystallising the sample (B). Crystallisation was performed by heating the powder scraped from a coated sample to 1000°C for 24 hours.

Coating samples for phase identification and quantification were collected by scraping the deposited layer of HA from Ti6Al4V coupons (see Figure 7). The XRD pattern show a slight bump at 2θ ~30.25, which indicates the formation of a small amount (6% w/w) of beta tricalcium phosphate (β-TCP). No evidence was found in XRD for the formation of other calcium phosphate phases or of calcium oxide. The crystallinity ratio was calculated to be 70%, calculated from the relative integral of 10 peaks before and after sintering at 1000°C. Table 1 summarises the results of the physiochemical characterisation of the HA coatings. These results are compared to the requirements set by ISO 13779-2 for HA coatings.

**Table 1.** Material characterisation of HA coatings compared to requirements set by ISO-13779-2.

Metric	ISO standard (ISO 13779-2)	Result	Compliance to ISO 13779-2 Yes/No
Phase purity – CaP phases other than HA	≤30%	6% β-TCP 0% other phases	Yes
Phase purity – presence of CaO	≤5%	0%	Yes
Crystallinity ratio	≥45%	70%	Yes
Adhesion strength	≥15 MPa (average) ≥10 MPa (all samples)	80 ± 2 MPa	Yes

### Adhesion strength tests on HA-coated Ti6Al4V cylinders

**Figure 8.**
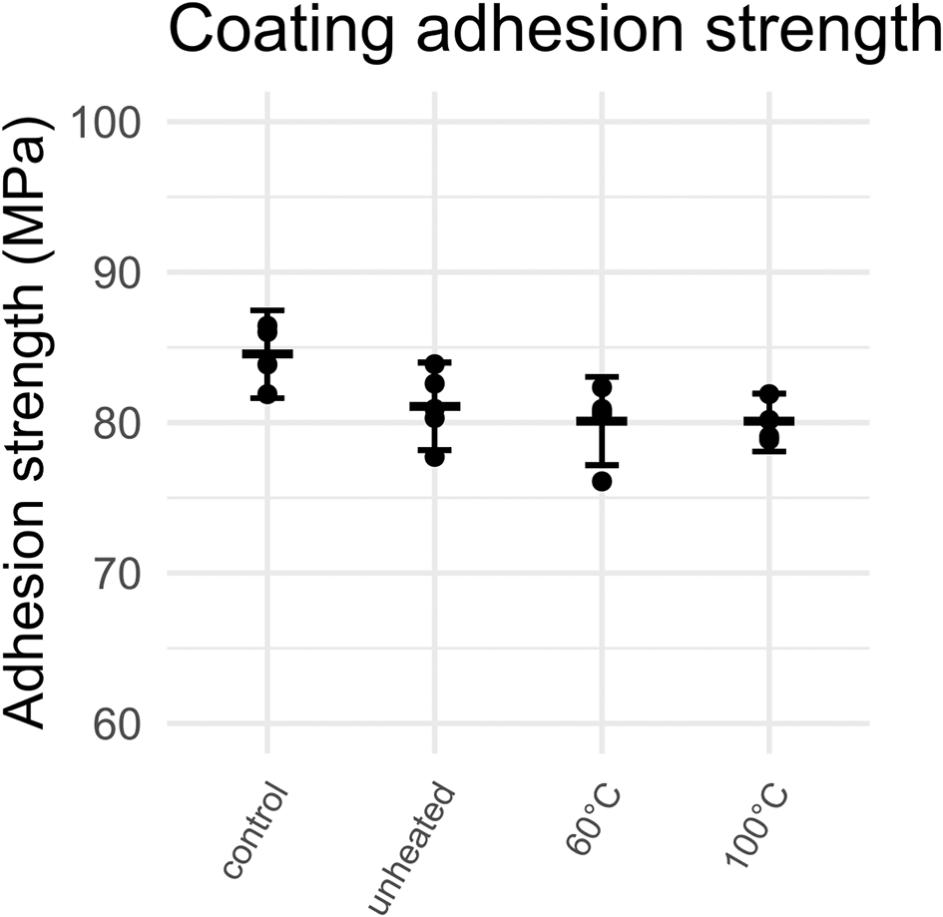
Adhesion strength of HA coatings heated to different temperatures. Error bars represent mean and 95% CI.

The adhesion strengths of Ti6Al4V cylinders are shown in Figure 8. Most failure modes were of type A for uncoated control samples and C for coated samples. However, two controls exhibited a mixed A/B mode of failure, most likely due to incorrect levelling of the surface of the cylinders. One sample from the 100°C group was rejected after a human error made it unsuitable for testing. After omission of this sample from further analysis, a sensitivity analysis was performed, showing that all results of this study remained similar and the conclusions did not change. On average, the strength of coated samples and uncoated controls was 80 MPa and 85 MPa, respectively. This indicates that the FM-1000 glue used for the experiment was stronger than the adhesion strength of the HA coating, making it suitable for the experiment. It is important that the glue is stronger than the coating in order to properly distinguish between different modes of failure. No difference in adhesion strength was found between coated samples based on their heating temperature.

### Bone cement – adhesion of cemented implants

**Figure 9.**
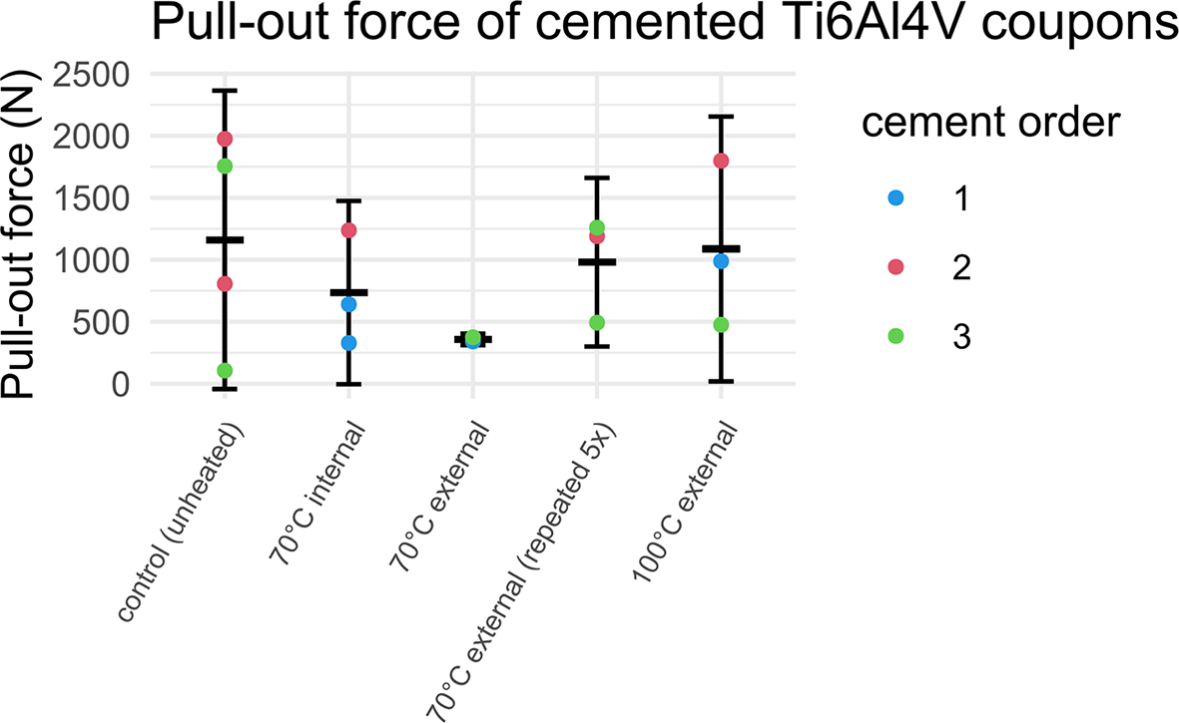
Pull-out force for cemented Ti6Al4V coupons heated to different temperatures. Error bars represent mean and 95% CI.

**Figure 10.**
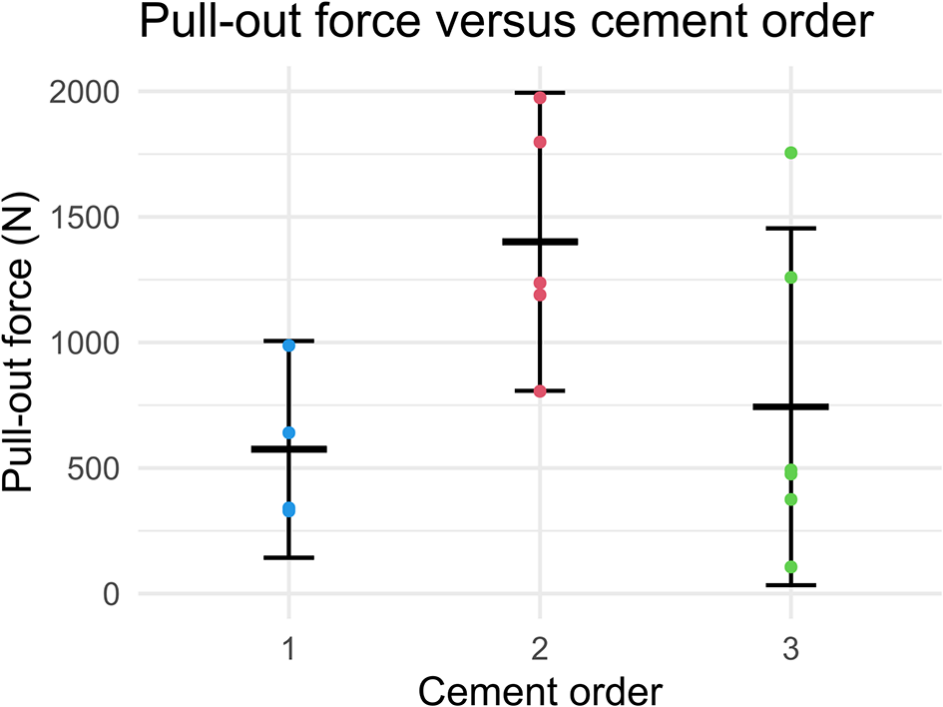
Pull-out force of Ti6Al4V coupons grouped by the order of cement application. Error bars represent mean and 95% CI.

The results of the post-heating pull-out experiments are shown in Figures 9 and 10. Detailed data on the pull-out force of individual samples can be found in the Underlying data. ^21^ Because there is no proper standard for measuring the pull-out strength of PMMA bone cement, the procedure for measuring the adhesion strength for HA coatings was followed as closely as possible.

No clear relationship between the heating temperature and the pull-out force was observed (see Figure 9). Likewise, there was no significant difference between controls and grouped heated samples. However, there appeared to be an association between the order of cementation and the resultant pull-out force (see Figure 10).

### *In-situ* induction heating of cemented Ti6Al4V coupons under tension

The internal temperature that caused failure for re-inserted samples loaded with a weight of 2.5 kg was 83°C with a SD of 11°C. One sample was excluded because the titanium coupon did not disengage from the cement at 150°C. Detailed data on the pull-out force of individual samples can be found in the Underlying data. ^21^

## Discussion

The results of this study show that the adhesion strength of HA coatings is unaffected by heating to 60°C or 100°C. Similarly, no relationship was established between the pull-out force and the heating temperature of cemented coupons. However, there was an association of the cementation order and the pull-out strength. Furthermore, it is clear that heating bone cement above its glass transition temperature results in a reversible softening that could potentially allow an implant to loosen if force is applied during active heating.

### HA coatings – adhesion strength of uncemented implants

The physiochemical requirements for HA coatings in medical devices is outlined by ISO 13779-2. ^14^ The minimum adhesion strength for any given coating is 10 MPa, with an average no lower than 15 MPa. Furthermore, ISO 13779-2 stipulates that the crystallinity ratio of an HA coating must be above 45% by weight and can contain no more than 30% of other calcium phosphate phases and no more than 5% calcium oxide. Based on these requirements, the coatings made in this study adhere to the ISO standard.

The XRD pattern of the coated sample indicates some loss of crystallinity and the formation of a small amount of β-TCP. β-TCP, with chemical formula Ca _3_(PO _4_) _2_, is a different calcium phosphate phase known to be more bioactive than HA due to its higher solubility. HA can convert into β-TCP when subjected to temperatures above 1000°C. ^22^ The formation of β-TCP in the HA coating (6%) can be explained by the high temperatures experienced by the coating powder during the plasma spray process, but remains below the upper limit (30%) specified by ISO 13779-2. Moreover, the calculated crystallinity ratio (70%) is within the requirements (45%) set by ISO 13779-2. Surface roughness measurements and trace element analysis are also mentioned in ISO 13779-2; however, these measurements were not performed in this study as they fall outside the scope of this investigation. It is not expected that these parameters influence the change in adhesion strength after NCIH treatment.

The difference between uncoated control samples and coated samples was 4.17 MPa, which is slightly smaller than the requirement of 5 MPa set by ISO. The ISO requirement is set to be able to properly distinguish between failure of the glue and failure of the coating. Nevertheless, a statistically significant difference was found between controls and coated samples. The coating adhesion strength for the HA coatings tested in this study (average of 80 MPa across all groups) is well above the requirement of 10MPa set by ISO 13779-2. That means these coatings would be acceptable for orthopaedic purposes. The fact that no significant change in adhesion strength was found after NCIH is unsurprising, as the coating particles are already exposed to extremely high temperatures during the plasma spraying process. This study shows that there is no increased likelihood of implant failure due to coating delamination when using NCIH on coated implants.

### Bone cement – adhesion of cemented implants

The glass transition temperature (T _glass_) is a property of amorphous solid materials. Below T _glass_, the solid is in a ‘glassy’ state, i.e., rigid and inflexible. Above T _glass_, the material becomes flexible and rubbery. T _glass_ can be either below room temperature (e.g., rubber) or above room temperature (e.g., polystyrene). For fresh, dry bone cement, T _glass_ is in a range of 80-100°C, meaning bone cement is in its ‘glassy’ state under physiological conditions, which gives it excellent properties for bone fixation. ^11^ When the temperature exceeds T _glass_, bone cement is known to weaken considerably, which could compromise implant fixation during NCIH treatment. Interestingly, this phenomenon was used to facilitate removal of bone cement in previous research. ^23^ As the temperatures intended for NCIH treatment (60-80°C) are close to the T _glass_ of bone cement, it is important to establish the impact of NCIH on implant fixation. In order to mimic the clinical situation as closely as possible, the cemented samples were prepared by an orthopaedic surgeon (BP).

The external temperature was recorded to reach up to 400°C in the group where the internal temperature was made to reach 70°C. Nevertheless, no noticeable weakening of bone cement was found even after heating to these extreme temperatures. Clearly, these extreme conditions exceed those that will be used in the clinic. Nevertheless, no permanent weakening of the cement was observed when the cement was allowed to cool down before attempting to remove the coupons, indicating that the mechanical properties of bone cement are not irreversibly compromised upon heating with NCIH. It should be noted that the surface characteristics of the medial-grade Ti6Al4V used in this study have been described in detail in previous research. ^13^ Generally, the surface finish of the coupons is much smoother than that of most commercial implants. ^24^ As such, it is expected that the grip strength reported here underestimates the clinical situation.

Despite the lack of evidence of weakening of the cement after heating, there was considerable spread in the measured pull-out force, with pull-out forces varying from 330 to 1970N. This spread could partially be explained by the timing of the fixation of the Ti6Al4V coupons relative to the mixing of the precursors. From each batch of bone cement, three samples were cemented in place, with ~2.5 minutes in between each sample. Samples that were cemented immediately after mixing were found to be weakest, while the second sample was the strongest. There was an increasing trend in the variability in the pull-out strength going from the first to the third sample. This increased variability is likely due to variations in the speed at which the samples were prepared. The importance of the timing of implant cementation is in line with previous research and the experience of Orthopaedic surgeons. ^20^ Other sources of variability most likely include variations in the angle of the sample and cementation technique. However, these variations are expected to average out due to the random allocation of the sample between the groups.

When force was applied to the cemented coupons during active heating, it became clear that the cement became slightly pliable upon heating to >80°C, allowing the coupon was able to slip out. This point likely corresponds to the glass transition temperature of the bone cement, which was reported to fall around 90°C for dry cement samples of this brand. ^11^ The loosening temperatures observed in this study are above the intended temperatures of NCIH (70°C or less); therefore, it is not expected that NCIH treatment will lead to implant loosening. Nevertheless, as T _glass_ differs between cement brands and the age of the cement, it would be prudent to stabilise the patient’s limb during NCIH until the treatment is finished, if that patient uses a cemented prosthesis. ^11^ As this study shows that the weakening of bone cement is non-permanent, there should be no permanent effect on the prosthesis fixation after the treatment is over even if T _glass_ is exceeded for a short period of time.

### Limitations & Strengths

There are some limitations to this study that should be considered. Most importantly, it should be noted that all testing samples were exposed to air during the heating experiments and strength tests, as opposed to a biological environment. One consideration is that the properties of PMMA cement and HA coatings could change over time *in vivo* due to degradation by physical and biological processes. Specifically, T _glass_ for PMMA cement tends to decrease with time under biological conditions. ^11^ Thus, it is possible that T _glass_ is exceeded by NCIH heating in patients with older prostheses. A second limitation of our study is that the rate of heating and heat dissipation are different for samples in an ambient environment, compared to *in vivo* conditions. It is expected that implanted metal will heat more slowly, as surrounding tissue acts as a heat sink. While this means the temperature can be better controlled *in vivo*, it also means that the thermal dose required to reach the desired temperature will be higher. To mitigate this potential risks the temperatures used in study are a ‘worst case scenario’ that exceeds those desired in NCIH treatment.

Despite these limitations, this study is able to assess the impact of NCIH on the fixation of HA implant coatings and PMMA bone cement with a high degree of fidelity. Samples were prepared using industrial equipment and medical-grade materials. Furthermore, the coating adhesion tests were conducted using industry-standard tests published by ISO. Pull-out experiments on cemented coupons were adapted from those same tests to maximise reliability of the data. Based on our results, NCIH does not affect the fixation of uncemented (HA-coated) and cemented implants.

## Conclusion

It was shown that induced temperatures up to 100°C do not decrease the adhesion strength of HA coatings, as tested using certified methods set by ISO 13779-2. In contrast to the HA coatings, bone cement was found to weaken above 80°C, allowing the implant material to be pulled out. This weakening effect is most likely associated with the glass transition temperature of the material. This weakening was shown to be reversible, as there was no significant difference between the fixation strength of any of the groups after cooling. A considerable variability in the fixation strength was found among different samples, which could partially be explained by differences in the timing of the cementation.

### Acknowledgements and affiliations

This publication is part of the project DARTBAC (with project number NWA.1292.19.354) of the research programme NWA-ORC which is (partly) financed by the Dutch Research Council (NWO).

This article was written in collaboration with CAM Bioceramics B.V., situated in Leiden, The Netherlands. CAM Bioceramics B.V. is a contract development and manufacturing organisation of Orthobiologic Calcium Phosphates.

The authors would like to acknowledge the work of Larbi Douz, Rob Bockhorst and Marco Azier from CAM Bioceramics B.V. for their contribution in the preparation of the HA coatings and measurement of the coating adhesion strength.

## Ethics and consent

Ethical approval and Consent not required.

## Data Availability

Harvard Dataverse. Supplementary data - Effect of non-contact induction heating on HA coatings and bone cement: an ex vivo study. DOI: https://doi.org/10.7910/DVN/C1AWLO. ^21^ This project contains the following underlying data:
• Supplementary data.docx. (document containing information such as adhesion strength, pull-out force, coating thickness, and final temperature reached for each sample). Data is available under the terms of the Create Commons “Attribution 4.0 International” data waiver (CC-BY 4.0 Attribution-Only).
