# Supplementary material for: Effect of non-contact induction heating on HA coatings and bone cement, an ex vivo study
Source: F1000Res. 2024 Oct 22;13:443. Originally published 2024 May 3. [Version 2] doi: 10.12688/f1000research.148225.2 (PMC11757918; doi:10.12688/f1000research.148225.2)
Supplement: Peer review history

# Reviewer report: Reviewer response for version 2

**DOI:** 10.5256/f1000research.172963.r357657

**Published:** 2025-01-23

**Authors:** Kimberly M. Brothers

**Affiliations:** 1 University of Pittsburgh, Pittsburgh, Pennsylvania, USA

The authors do nice work characterizing the effects heating on HA coated implants and PMMA and how this may affect implant retention. For future studies, it will be very interesting to characterize how this method can reduce reduce multi-drug resistant biofilms and other pathogens. Looking at the reviewer comments, it looks like the authors have addressed all concerns.

 Strengths

1. Introduction covers all relevant aspects of the field.
2. Methods section provides good detail on how the experiments were conducted including the use of Figures.
3. Discussion section is relevant to the findings presented including mentioning limitations of the study.

Weaknesses

1. Bacteria names in introduction should be in italics.
2. The results section is rather brief. More descriptive findings for the headings for each section would be useful.
3. It would be useful to add a paragraph for future studies including if this will be tested against bacterial biofilms and multi-drug resistant pathogens.

Is the work clearly and accurately presented and does it cite the current literature?

Yes

If applicable, is the statistical analysis and its interpretation appropriate?

Yes

Are all the source data underlying the results available to ensure full reproducibility?

Yes

Is the study design appropriate and is the work technically sound?

Yes

Are the conclusions drawn adequately supported by the results?

Yes

Are sufficient details of methods and analysis provided to allow replication by others?

Yes

Reviewer Expertise:

Biofilms and infectious disease

I confirm that I have read this submission and believe that I have an appropriate level of expertise to confirm that it is of an acceptable scientific standard.

# Reviewer report: Reviewer response for version 2

**DOI:** 10.5256/f1000research.172963.r333817

**Published:** 2024-12-02

**Authors:** Joeri Kok

**Affiliations:** 1 Eindhoven University of Technology, Eindhoven, The Netherlands

I thank the authors for revising their article. I have no further comments.

Is the work clearly and accurately presented and does it cite the current literature?

Yes

If applicable, is the statistical analysis and its interpretation appropriate?

No

Are all the source data underlying the results available to ensure full reproducibility?

Yes

Is the study design appropriate and is the work technically sound?

Yes

Are the conclusions drawn adequately supported by the results?

Partly

Are sufficient details of methods and analysis provided to allow replication by others?

Partly

Reviewer Expertise:

Orthopaedic biomechanics; Numerical analysis; Medical image analysis

I confirm that I have read this submission and believe that I have an appropriate level of expertise to confirm that it is of an acceptable scientific standard.

# Reviewer report: Reviewer response for version 1

**DOI:** 10.5256/f1000research.162511.r319607

**Published:** 2024-09-11

**Authors:** Joeri Kok

**Affiliations:** 1 Eindhoven University of Technology, Eindhoven, The Netherlands

The work that the authors present provides novel insight on the adhesion of (bio)materials hydroxyapatite and PMMA to Ti6Al4V during induction heating. Overall the manuscript is well-written. The introduction and aim of the work is clear and the methodology largely follows ISO standards making it reliable and repeatable. I do have some remaining questions regarding the strength of the study.

 1. The number of samples per group severely hinders drawing any hard conclusions. First of all, the number of samples per group is not mentioned in the methods and is only clear from the supplementary material. Secondly, I believe that it is inappropriate to use parametric tests that assume normality for the statistics. With low sample numbers non-parametric tests should be used because tests for normality cannot be relied upon. In the case of the tests with bone cement I would even like to suggest to remove the statistics completely and stick to only describing the observations. With groups of 2-4 samples standard deviations don't mean much and statistical significance cannot be reached, thus potentially giving readers a false impression of similarity between groups.   

 2. For the adhesion experiments with HA, the supplementary material shows that the failure mode is always 'mode C'. I understand the difference between the modes but how are the failure modes determined? Can you visually see the difference between failure mode C and D, or a mix? It would be valuable to include photos in a figure of each observed failure mode.

 3. For the in-situ induction heating tests. How are the coupons exactly reinserted using a vise? Why are they reinserted in the same cement and not put into new cement? Does the reinsertion not make the connection dependent on the previous heating methods?

 4. Based on figure 5: Overall this part of the study is not very well explained. What is on the horizontal axis? If this is the time, why not start at t=0? Why was manual activation and deactivation of the heating device required? Why such short intervals? Does the speed of heating influence the failure temperature?

 5. On average a difference between adhesion of the glue and the HA coating is shown. I already somewhat doubt the significance of this measure, additionally, figure 8 shows overlap between control and the other groups. Regardless of the statistical difference this suggests that there will be samples where the adhesion/cohesion of the glue will fail before that of the HA coating. Coming back to comment 2, I'm very curious about how the failure was observed and defined for each individual sample.

 6. The results about the batches are interesting, but they come somewhat out of the blue. I suggest explaining this also in the methods with an estimate of the variation in time between mixing and inserting the coupons.

Is the work clearly and accurately presented and does it cite the current literature?

Yes

If applicable, is the statistical analysis and its interpretation appropriate?

No

Are all the source data underlying the results available to ensure full reproducibility?

Yes

Is the study design appropriate and is the work technically sound?

Yes

Are the conclusions drawn adequately supported by the results?

Partly

Are sufficient details of methods and analysis provided to allow replication by others?

Partly

Reviewer Expertise:

Orthopaedic biomechanics; Numerical analysis; Medical image analysis

I confirm that I have read this submission and believe that I have an appropriate level of expertise to confirm that it is of an acceptable scientific standard, however I have significant reservations, as outlined above.

# Response

**Published:** 2024-10-03

**Authors:** Robert Kamphof

**Affiliations:** Orthopaedics, Leiden University Medical Center, Leiden, The Netherlands

Dear reviewer,

 Firstly, we would like to thank you for providing feedback so we can improve our article. We have made some changes to our article based on your feedback, and we would like to address some of your comments here.

1. We agree that the number of replicates per experimental condition was unclear in the first version our article, and we have adapted the methods section accordingly. We have also made some changes to clarify our workflow.
  Regarding the statistics, we fully agree with your assessment. Using parametric tests was not suitable for our data set. In general, there is a growing resistance against the (wrongful) use of p-values  (e.g. “Scientists rise up against statistical significance”, https://doi.org/10.1038/d41586-019-00857-9). Therefore, we have chosen to remove statistical tests from our manuscript, focusing instead the observed values. Moreover, we have changed the error bars in our images to represent the 95% CI instead of the standard deviation, and we have clarified this in the methods and the figure captions.
2. Indeed for the HA coated experimental groups, the mode of failure was always C, indicating that the adhesive strength of the coating was lower than the cohesive strength. The failure mode is determined by visual inspection of the fracture plane after failure. Failure mode D, indicating cohesive failure of the coating, would be visible by the presence of HA on both sides of the fracture plane. This was not the case in our experiments; instead, the coating was always stripped completely from the substrate by the glue. We have added images of the different failure modes in the supplementary data.
3. Thank you for this excellent question. The coupons were re-inserted into their respective cement constructs by pressing them into the slot in the cement as far as possible by hand, then opening a manual vise and closing it onto the construct until the coupon occupied its original position.
  The reason why we opted to re-use the constructs was due to the large variation in adhesion strength of these constructs. If we used fresh ones, their adhesion strength would be unknown, and it would be difficult to estimate the effect of heating in situ.
  As a control experiment, we have repeated our first experiment on re-inserted coupons, and found that the resulting adhesion strength remained relatively constant even when the coupon was re-inserted three or four times. This shows us that the observed adhesion strength under room temperature conditions is mostly due to friction, and that the cement is not permanently damaged by failure.
4. We used pulsed heating to achieve to ensure homogenous heating of the coupons. Inhomogeneities in the magnetic field or the Ti6Al4V-PMMA constructs could lead to uneven heating and the formation of hot spots if constant heating was applied. By applying a heating duty cycle, any hot spots had time to diffuse. Secondly, duty cycling reduced the change of overheating in the induction coil itself. We have made some changes to the manuscript to clarify these points, and we have removed Figure 5 since we agree it raises more questions than it answers.
5. It is true that the difference between the measured strength of the coatings differed only slightly from the strength of the glue. Ideally, a larger difference would be observed to properly distinguish between the failure modes (NB: ISO-13779-2 demands an average difference of 5MPa between the glue and the samples, which we achieved, if only narrowly). However, the strength of the control does not change the main findings of our study, namely that that no difference was observed between the coated groups heated at different temperatures.
6.  Thank you for your feedback on this. We have made some changes to the manuscript. We estimate that the time between the cementation between each sample was about 2.5 minutes. Naturally, there was some variation in this time, which explains the increasing spread in the samples with increasing cement order.

We would like to thank you again for your feedback. We hope to have answered your questions and remarks in a sufficient manner and that you are happy with the changes made to the document.

# Reviewer report: Reviewer response for version 1

**DOI:** 10.5256/f1000research.162511.r298322

**Published:** 2024-08-27

**Authors:** Govinda Kapusetti

**Affiliations:** 1 National Institute of Pharmaceutical Education and Research (NIPER), Kolkata, India

The study was interesting but need to clarify the following issues before indexing;

1. The study found that NCIH up to 100°C did not significantly impact the adhesion strength of HA coatings. This suggests that the use of NCIH on HA-coated implants is feasible without risking coating delamination, which is crucial for maintaining the integrity of the implant.
2. The study also investigated the impact of NCIH on acrylic bone cement. The bone cement softened as the temperature approached and exceeded the glass transition temperature (around 83°C). However, this softening was reversible, and no significant loss of fixation strength was detected once the cement cooled down.
3. Verify the statement that PMMA is more thermally stable than HA, and correct it.
4. The study focuses on the effects of NCIH at temperatures up to 100°C. Consider conducting additional experiments at slightly higher temperatures (e.g., 110°C or 120°C) to determine the upper limit at which HA coatings and bone cement maintain their structural integrity. This will provide a more comprehensive understanding of the safe operating range for NCIH.
5. The study appears to focus on short-term heating effects. It would be beneficial to perform experiments that expose samples to prolonged heating durations to simulate potential clinical scenarios where longer exposure might be necessary. This will help determine if there are cumulative effects that could compromise material properties over time.
6. Perform chemical stability tests to determine if NCIH causes any degradation or chemical alterations in HA coatings or bone cement. For instance, Fourier-transform infrared spectroscopy (FTIR) or Raman spectroscopy could be used to detect any changes in the chemical bonds or phase transitions.
7. Given that NCIH is being considered for clinical applications, conducting in vitro biocompatibility studies on materials post-NCIH treatment would be crucial. This could involve cell viability assays, such as MTT or Live/Dead assays, using osteoblasts or fibroblasts to assess if the heated materials induce any cytotoxic effects.
8. Thermal imaging or infrared thermography is employed to visualize and quantify the heat distribution across the HA coatings and bone cement during NCIH. This will help ensure that the heating is uniform and does not create localized hotspots that could potentially damage the material or surrounding tissues.
9. The study has strong potential for clinical impact. By exploring the feasibility of using NCIH in a medical context, the research opens up new possibilities for non-invasive treatment of implant-related complications, which could significantly benefit patient outcomes.
10. In conclusion, this paper presents a well-executed and innovative study that significantly contributes to the understanding of non-contact induction heating (NCIH) and its effects on HA coatings and bone cement. The research is grounded in a strong rationale, and the findings have important implications for both materials science and clinical applications. While there are areas for further exploration, particularly regarding long-term effects and practical implementation, the study provides a solid foundation for future research. It has the potential to influence advancements in the treatment of implant-related complications.

Is the work clearly and accurately presented and does it cite the current literature?

Yes

If applicable, is the statistical analysis and its interpretation appropriate?

Partly

Are all the source data underlying the results available to ensure full reproducibility?

Yes

Is the study design appropriate and is the work technically sound?

Yes

Are the conclusions drawn adequately supported by the results?

Partly

Are sufficient details of methods and analysis provided to allow replication by others?

No

Reviewer Expertise:

Orthopedic materials, smart devices, and martials, theranostics

I confirm that I have read this submission and believe that I have an appropriate level of expertise to confirm that it is of an acceptable scientific standard, however I have significant reservations, as outlined above.

# Response

**Published:** 2024-08-27

**Authors:** Robert Kamphof

**Affiliations:** Orthopaedics, Leiden University Medical Center, Leiden, The Netherlands

Dear reviewer,

 I would like to thank you for reading our article and for providing your suggestions for improvement. Let us respond to your feedback on a point-by-point basis.

**1.** This is correct. Our study has indeed found that HA coatings maintain their mechanical stability when heated up to 100°C using NCIH.

**2.**This is also correct. The glass transition temperature is a transition point where a polymer transforms from a solid and brittle ‘glassy’ state to a soft and malleable ‘rubbery’ state. The value of the glass transition temperature is dependent both on the chemical composition of the polymer as well as its chain length.

**3.** My apologies, but we cannot find the section in the manuscript where we write that PMMA is more thermally stable than HA. Could you point us towards the relevant section, so we can revise it? We would like to amend that section to avoid confusion.

**4.** We do not believe that there is an added value in performing experiments at higher temperatures for this study, as the maximum temperature of NCIH is constrained because to 100°C of its intended use in a biological (i.e., aqueous) environment. Having said that, we think more fundamental research on the thermal properties of HA and PMMA, such as TG/DSC analysis, would be interesting and insightful.

**5-7**. Thank you for your suggestions for further research. While we consider these suggestions very interesting, and we would like to see these questions answered in the future, we consider them to be outside the scope of our current study. We intended to answer the research question: ‘does NCIH of Ti6Al4V weaken the fixation of HA coatings or PMMA bone cement?’. We hope you agree with our assessment.

**8.** Thank you for your praise. We used a thermal camera to follow the temperature changes of the HA coated Ti6Al4V cylinder and we can confirm that no hotspots formed during heating, and that heating  was uniform across the circular surface of the cylinder. The heating of Ti6Al4V coupons using NCIH was previously described in the paper: ‘Induction heating for eradicating Staphylococcus epidermidis from biofilm’ ( https://doi.org/10.1302/2046-3758.94.BJR-2019-0274.R1). We intend to publish more research on NCIH, including more thermal images, in the near future.

**9-10.** Thank you for your kind feedback. We hope that our research can help in developing NCIH technology further so it can be safely and effectively employed in a clinical setting.

 Thank you again for your kind feedback. I hope you consider all your questions and concerns fully addressed by this response. If you have any further questions, please let us know.
